# Modular Model of Neuronal Activity That Captures the Dynamics of Main Molecular Targets of Antiepileptic Drugs

**DOI:** 10.3390/ijms27010490

**Published:** 2026-01-03

**Authors:** Pavel Y. Kondrakhin, Fedor A. Kolpakov

**Affiliations:** Department of Computational Biology, Scientific Center for Genetics and Life Sciences, Sirius University of Science and Technology, 354340 Sirius, Russia; kolpakov.fa@talantiuspeh.ru

**Keywords:** mathematical model, epilepsy, seizures, tripartite synapse, biophysical neuron model, antiepileptic drugs, BioUML

## Abstract

This paper presents a modular mathematical model of neuronal activity, designed to simulate the dynamics of main molecular targets of antiepileptic drugs and their pharmacological effects. The model was developed based on several existing synaptic transmission models that capture cellular processes crucial to the pathology of epilepsy. It incorporates the primary molecular mechanisms involved in regulating excitation and inhibition within the neural network. Special attention is given to the dynamics of ion currents (Na^+^, K^+^, Ca^2+^), receptors (AMPA, NMDA, GABA_A_, GABA_B_ and mGlu), and neurotransmitters (glutamate and GABA). Examples of simulations illustrating the inhibitory effects on synaptic transmission are provided. The numerical results are consistent with experimental data reported in the literature.

## 1. Introduction

Epilepsy is a chronic neurological disorder characterized by a predisposition to spontaneous seizures, often accompanied by comorbidities such as anxiety, depression, and cognitive impairment [1]. In the brain of a person with epilepsy, excitatory activity dominates over inhibitory one, leading groups of neurons to synchronously produce powerful electrical discharges. These discharges can spread to other parts of the nervous system, triggering seizures. Seizures manifest as brief episodes of altered behavior, which can include symptoms such as loss of consciousness, convulsions, or sudden stiffening of the body [2].

The causes of epilepsy are diverse, including mutations, metabolic disorders, head injuries, infections, and other factors that impair brain function [3]. Treatment typically focuses on symptom suppression through antiepileptic drugs (AEDs; also termed antiseizure medications), but in severe cases, surgical intervention may be necessary to address the underlying causes of epileptic activity. However, the high toxicity of AEDs complicates the optimization of drug therapy strategies [4]. Despite the availability of over 30 antiepileptic drugs with various mechanisms of action, approximately one-third of patients are drug-resistant [5].

To enhance understanding of the mechanisms underlying epilepsy, mathematical models have been developed [6]. Such models are valuable tools for validating hypotheses, as they allow for detailed examination of neuronal dynamics under controlled conditions. Using computational modeling to study the mechanisms of neuronal response to epilepsy drug therapy can enhance general understanding of this complex disease, improve treatment efficacy, and reduce the risk of side effects.

There are three main approaches to brain modeling: nanoscopic (cellular), which focuses on individual neurons; microscopic (population), which models interactions among neuron populations; and mesoscopic (regional), which examines entire brain regions. The microscopic approach provides detailed insights into neural network dynamics [7,8]. However, simulating neuronal populations, which often involve tens of thousands of neurons, results in high computational complexity, limiting the practical applicability of this approach. Mesoscopic models can generate accurate electrophysiological brain signals, such as electroencephalogram signals, that are consistent with experimental recordings [9,10]. However, these models are often based on purely mathematical formulations, lacking direct biological interpretation, which complicates the validation of results. On the other hand, nanoscopic models simulate biophysical processes occurring within individual neurons, including receptor dynamics, intracellular signaling pathways, and molecular interaction networks, as well as their synaptic interactions, and therefore, in most cases, provide strong biological significance [11,12,13]. Although they are not capable of reproducing whole-brain electrophysiological signals, these models capture detailed dynamics of cellular processes and generate signals that can be experimentally validated in vitro.

In addition to classification by the level of system organization, mathematical models of neuronal activity can be broadly categorized into phenomenological and biophysical models. Phenomenological models describe neuronal dynamics using simplified mathematical representations with relatively few variables and parameters [9,10,14,15]. These models effectively capture general patterns of neural activity and can reproduce key features observed in experimental conditions. However, their abstract nature limits direct biological interpretation, which limits their applicability for studying the detailed mechanisms underlying molecular interactions. Biophysical models, in contrast, incorporate detailed descriptions of ion channel kinetics, neurotransmitter dynamics, and synaptic transmission [11,12,13,16,17,18,19]. By explicitly modeling the underlying cellular processes, these models provide a high degree of biological interpretability and allow for direct comparisons with experimental data. However, this increased biological realism comes at the cost of higher computational complexity and a greater number of parameters. Typically, phenomenological models are used in mesoscopic and microscopic approaches, whereas biophysical models are predominantly applied at the nanoscopic level.

Consequently, nanoscopic biophysical models are particularly well-suited for simulating the dynamics of molecular targets of AEDs, which is the primary focus of this study.

Computational biology methods have developed very rapidly in recent years, and breakthroughs in neuronal activity modeling have enabled direct correspondences between experimentally measurable quantities and simulated biophysical processes, such as changes in ion concentrations and membrane potential oscillations [20,21,22]. This capability facilitates biologically meaningful modeling of the biophysical pathways targeted by AEDs, thereby simplifying the optimization of drug therapy.

The objective of this study was to develop a mathematical model of neuronal activity that accurately describes the functioning of neurons, taking into account the dynamics of the main molecular targets of AEDs. To accomplish this, we reproduced results from several existing synaptic transmission models on the BioUML (Biological Universal Modeling Language) platform, focusing on key cellular processes crucial in the pathology of epilepsy. Based on reproduced models, we constructed a modular model.

The paper is organized as follows. Section 2 presents the main results of the study. It first characterizes the main molecular targets of antiepileptic drugs, whose dynamics were considered in the model’s development, and formulates the corresponding requirements for the model. This is followed by a detailed description of the modular structure of the model and its main equations. The section then presents the results of numerical simulations, including the dynamics of neuronal and astrocytic activity, ion and neurotransmitter concentrations, demonstration of interneuron-mediated inhibition, and an example of drug effect simulation, with comparisons to experimental data from the literature. Section 3 discusses the obtained results and outlines the limitations and potential extensions of the model. Section 4 briefly describes the features of the BioUML software used for building the modular model and performing numerical calculations. Section 5 summarizes the main conclusions of the study. Appendix A, Appendix B, Appendix C, Appendix D and Appendix E provide a detailed description of all the model equations, as well as the parameter values and initial conditions for the variables.

## 2. Results

### 2.1. Main Molecular Targets of Antiepileptic Drugs

The first-line treatment for epilepsy typically involves AEDs, which symptomatically suppress seizures. Their mechanisms of action are aimed at regulating synaptic activity, modifying the intrinsic excitability properties of neurons and balancing inhibitory and excitatory neurotransmission, thereby reducing the probability of seizure occurrence.

**Figure 1:**
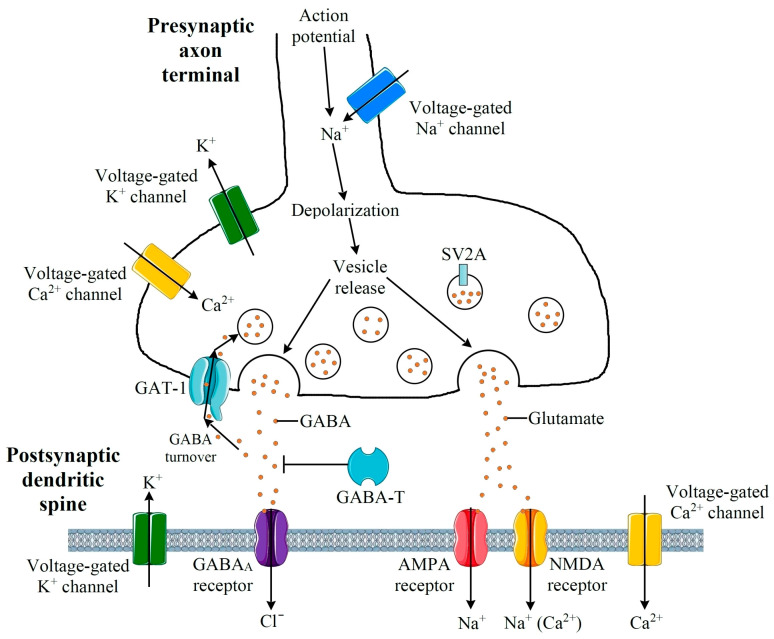
Schematic representation of the main molecular targets of AEDs, including voltage-gated Na^+^, K^+^, and Ca^2+^ channels, AMPA, NMDA, and GABA_A_ receptors, as well as synaptic vesicle protein 2A (SV2A), GABA transporter 1 (GAT-1), and GABA aminotransferase (GABA-T).

The actions of antiepileptic drugs are based on the effect on molecular targets that regulate neuronal activity. Main molecular targets of AEDs include voltage-gated sodium (Na^+^), potassium (K^+^) and calcium (Ca^2+^) channels; ionotropic receptors of a-amino-3-hydroxy-5-methyl-4-isoxazolepropionic acid (AMPA-Rs), N-methyl-D-aspartate (NMDA-Rs) and γ-aminobutyric acid (GABA_A_-Rs); as well as synaptic vesicle protein 2A (SV2A), GABA transporter 1 (GAT-1) and GABA aminotransferase (GABA-T) [23]. These targets are schematically shown in Figure 1.

In addition to GAT-1, which is predominantly expressed in neurons and acts as the main regulator of extracellular GABA in the hippocampus, there is also GAT-3, primarily expressed in glial cells, which plays a significant role in conditions of excessive neuronal activity [24]. The experimental observations indicate GAT-3 can release GABA from astrocytes into the synaptic space, contributing to astrocytic modulation of hyperexcitability [25,26]. Although no current antiepileptic drugs specifically target GAT-3, its activation leads to the inhibition of synaptic transmission [24]. Thus, GAT-3 dynamics are also of significant interest in modeling excessive synaptic activity.

Pharmacological action on primary targets of AEDs suppresses seizures through the following mechanisms:

- Activation of voltage-gated K^+^ channels or inhibition of voltage-gated Na^+^ and Ca^2+^ channels, leading to membrane hyperpolarization and thereby returning the neuron to its resting state;
- Enhancement of GABA-mediated inhibition via inhibition of the GAT-1 GABA transporter in neurons and astrocytes, inhibition of GABA transaminase, and activation of GABA_A_ receptors;
- Attenuation of synaptic excitation through inhibition of postsynaptic AMPA-Rs and NMDA-Rs;
- Direct modulation of synaptic release by activating presynaptic SV2A and inhibiting voltage-gated Ca^2+^ channels.

### 2.2. Requirements for the Modular Model

Based on the mechanisms of action of AEDs discussed above, the developed modular model of neuronal activity should account for the following:

- Na^+^, K^+^ and Ca^2+^ concentration dynamics, which are fundamental to the generation of action potentials (APs). Specifically, the model should incorporate voltage-gated channels for these ions.
- Dynamics of AMPA-Rs, NMDA-Rs and GABA-Rs, which are key regulators of excitatory and inhibitory synaptic transmission.
- Astrocytic regulation of synaptic cleft homeostasis, particularly the dynamics of the GAT-3.

These aspects have been incorporated into the model’s design.

### 2.3. Modular Model Structure

It is well established that glial cells, particularly astrocytes, play a crucial role in neurotransmission in addition to neurons [27]. Therefore, to accurately describe the mechanisms of neuronal activity, it is appropriate to consider tripartite (three-part) synapse models, which include not only the presynaptic and postsynaptic neurons but also perisynaptic astrocytes.

In our analysis of existing neuronal activity models, we focused on those within the tripartite synapse formalism.

Table 1 lists the neuronal activity models that were most relevant for our study and directly used in the development of the modular model. While numerous other models have been proposed in the literature, offering valuable insights into various aspects of neuronal dynamics, they were less suitable for direct integration into our framework. A broader discussion of these models and their relevance to our specific objectives is provided in Section 3.

**Figure 2:**
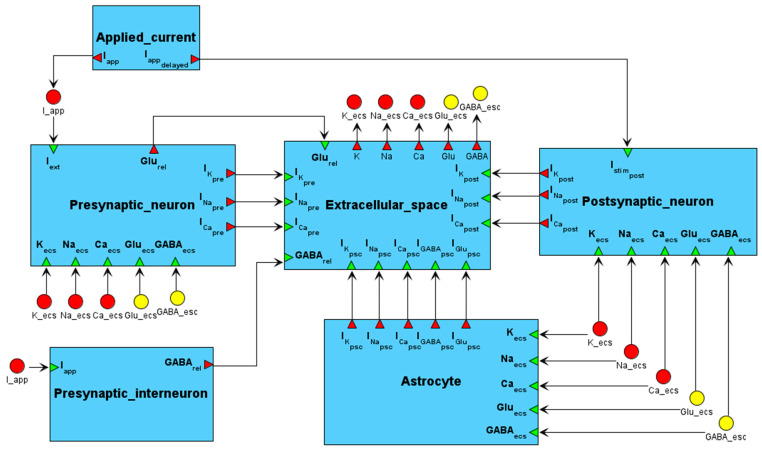
Modular structure of the model. The model consists of six interconnected modules representing the presynaptic and postsynaptic neurons, perisynaptic astrocyte, interneuron, extracellular space, and externally applied current. It captures glutamate release from the presynaptic neuron and GABA release from the interneuron, along with the regulation of ion concentrations and ionic currents across neuronal and astrocytic membranes.

The mathematical model of neuronal activity that captures the dynamics of main molecular targets of antiepileptic drugs was developed based on the principles of modular modeling of complex biological systems using the BioUML platform. The modular structure of the model in BioUML is shown in Figure 2.

The core of the model was based on [11] and was further extended to include the regulation of GABA and glutamate dynamics by astrocytes as detailed in [25]. Additionally, the model incorporated GABA_A_, GABA_B_, and metabotropic glutamate receptors (mGlu-Rs) [25,28], as well as the inhibitory effects of interneurons [12].

The BioUML implementation of the model consists of six modules: presynaptic and postsynaptic neurons, perisynaptic astrocyte, interneuron, extracellular space, and externally applied current. It includes 247 variables, 41 differential equations, 174 parameters, and 235 assignment operations. Hereafter, it is referred to as the modular model of neuronal activity.

The model’s modules are briefly described as follows:

1. **Presynaptic neuron** (Presynaptic_neuron): Module that describes the dynamics of the presynaptic axon terminal, incorporating voltage-gated ion channels, sodium-potassium and calcium ATPases (NKA and PMCA), GABA_A_-Rs involved in tonic astrocyte-mediated modulation, and external stimulus. It calculates the concentration of glutamate released into the extracellular space, with the release rate primarily determined by presynaptic calcium entry, while being modulated by the dynamics of mGlu-Rs and GABA_B_-Rs. Ion fluxes across the membrane are calculated as well.
2. **Postsynaptic neuron** (Postsynaptic_neuron): This module describes the dynamics of the postsynaptic dendritic spine, including voltage-gated ion channels, NKA, PMCA, AMPA-Rs, NMDA-Rs, as well as GABA_A_-Rs activated by astrocyte-mediated tonic GABA signaling, and a weak external stimulus. Ion fluxes across the membrane are also computed.
3. **Perisynaptic astrocyte** (Astrocyte): This module describes the dynamics of the astrocyte, including the excitatory amino acid transporter 2 (EAAT-2), the sodium-calcium exchanger (NCX), NKA, inwardly rectifying potassium channel (Kir4.1), and the GABA transporter 3 (GAT-3). Ion diffusion within the astrocytic process is considered, along with ion and neurotransmitter fluxes across the membrane.
4. **Interneuron** (Presynaptic_interneuron): This module calculates the inhibitory effect of the interneuron by calculating the release of GABA into the extracellular space. The excitation is initiated by an external current injection and mediated by the activation of voltage-gated ion channels.
5. **Extracellular space** (Extracellular_space): This module describes the dynamics of the synaptic cleft, where concentrations of ions and neurotransmitters are established based on the fluxes across the membranes of the adjacent cellular compartments. Ion diffusion is also considered.
6. **Externally applied electrical current** (Applied_current): This auxiliary module calculates stimulating currents for the presynaptic neuron, postsynaptic neuron, and interneuron.

### 2.4. Main Equations of the Modular Model

This section provides a brief overview of the main equations underlying the modular model of neuronal activity (Equations (1)–(24)). Detailed descriptions of all equations, parameter values, and initial conditions for the variables can be found in Appendix A, Appendix B, Appendix C, Appendix D and Appendix E.

The presynaptic and postsynaptic neurons, along with the interneuron, are modeled using the Hodgkin-Huxley-based description [29] for voltage-gated ion channels dynamics. The membrane potential of these cells is calculated in Equations (1)–(3) as follows:

(1) $$C_{pre}\frac{dV_{pre}}{dt}=I_{app}-\left(I_{Na,pre}+I_{K,pre}+I_{Ca,pre}+I_{L,pre}+I_{GABAA,pre}\right),$$

(2) $$C_{post}\frac{dV_{post}}{dt}=I_{app_{delayed}}-\left(I_{Na,post}+I_{K,post}+I_{Ca,post}+I_{L,post}+I_{GABAA,post}\right),$$

(3) $$C_{int}\frac{dV_{int}}{dt}=I_{app}-\left(I_{Na,int}+I_{K,int}+I_{L,int}\right),$$

where $V_{pre}$, $V_{post}$ and $V_{int}$ denote the membrane potentials of the presynaptic neuron, postsynaptic neuron and interneuron, respectively; C is the membrane capacitance; $I_{Na}$, $I_{K}$ and $I_{Ca}$ are the currents of Na^+^, K^+^ and Ca^2+^ ions across the membrane, respectively; $I_{L}$ is the leak current; $I_{app}$ and $I_{{app}_{delayed}}$ are the externally applied currents. $I_{app}$ is used to excite the presynaptic neuron and interneuron, while $I_{{app}_{delayed}}$ is required to remove the NMDA-Rs magnesium block. $I_{{app}_{delayed}}$ is introduced following the original synapse-astrocyte model [11] as a numerical surrogate for local postsynaptic depolarization in the absence of explicit somatic and dendritic morphology. This weak depolarizing current is applied with a 2 ms delay relative to the presynaptic neuron and has a much smaller amplitude, calculated to ensure that the postsynaptic neuron is excited as a result of presynaptic excitation, but that $I_{{app}_{delayed}}$ alone is insufficient to trigger postsynaptic neuron’s excitation.

Presynaptic membrane ionic currents are calculated considering voltage-gated ion channels, NKA, PMCA, and leak channels using Equations (4)–(6):

(4) $$I_{Na,pre}=I_{Na_{vg},pre}+3I_{NKA,pre}+I_{NaL,pre},$$

(5) $$I_{K,pre}=I_{K_{vg},pre}-2I_{NKA,pre}+I_{KL,pre},$$

(6) $$I_{Ca,pre}=I_{Ca_{vg},pre}+I_{PMCA,pre}+I_{CaL,pre},$$

where $I_{{Na}_{vg}}$, $I_{K_{vg}}$, $I_{{Ca}_{vg}}$ denote voltage-gated Na^+^, K^+^ and Ca^2+^ channels currents, respectively; $I_{NKA}$, $I_{PMCA}$ are NKA and PMCA generated currents; and $I_{NaL}$, $I_{KL}$, $I_{CaL}$ represent leak currents.

The presynaptic neuron also calculates the concentration of glutamate released into the synaptic cleft (Equation (7)), with its release probability primarily determined by presynaptic calcium entry and modulated by activation of mGlu-Rs and GABA_B_-Rs (Equation (8)), where the former increases [30] and the latter decreases it [31].

(7) $$Glu_{rel}=U\cdot T_{\max}\cdot RR,$$

(8) $$U=U_{0}+\left(1-U_{0}\right)r_{mGluR}-U_{0}r_{GABAB},$$

where ${Glu}_{rel}$ denotes the concentration of glutamate released from the presynaptic neuron vesicles per second; $U_{0}$ and U are the probabilities of glutamate release at rest and over time, respectively; RR is glutamate release rate; $T_{max}$ is glutamate concentration per vesicle; $r_{mGluR}$ and $r_{GABAB}$ are the fractions of activated mGlu-Rs and GABA_B_-Rs, respectively.

Ionic currents of the postsynaptic neuron (Equations (9)–(11)) additionally include AMPA-Rs and NMDA-Rs, which are necessary for successful synaptic transmission.

(9) $$I_{Na,post}=I_{Na_{vg},post}+3I_{NKA,post}+I_{AMPA,post}+I_{NMDA,post}+I_{NaL,post},$$

(10) $$I_{K,post}=I_{K_{vg},post}-2I_{NKA,post}-I_{NMDA,post}+I_{KL,post},$$

(11) $$I_{Ca,post}=I_{Ca_{vg},post}+I_{PMCA,post}+I_{NMDA,post}+I_{CaL,post},$$

where $I_{AMPA}$ and $I_{NMDA}$ are AMPA-Rs and NMDA-Rs generated currents, respectively.

The interneuron inhibits synaptic transmission by releasing GABA into the extracellular space, with its released concentration described by Equation (12). This process is modeled using the Tsodyks–Markram synaptic plasticity model [32], which accounts for the limited availability of synaptic resources and their recovery dynamics (Equations (13)–(15)).

(12) $$GABA_{rel}=n_{\nu }g_{\nu }E,$$

(13) $$\frac{dR}{dt}=\frac{I}{\tau_{rec}}-R\cdot \delta \left(t-t_{sp}\right),$$

(14) $$\frac{dE}{dt}=-\frac{E}{\tau_{inact}}+R\cdot \delta \left(t-t_{sp}\right),$$

(15) I=1−R−E.

Within this scheme ${GABA}_{rel}$ denotes the concentration of GABA released from the interneuron per second; R, E and I are the fractions of synaptic vesicles in the recovered, active and inactive states, respectively; $\tau_{rec}$ and $\tau_{inact}$ are vesicle recovery and inactivation time constants, respectively; $n_{v}$ is the number of docked vesicles; $g_{v}$ is the GABA concentration in single vesicle; δ is the Dirac delta function; $t_{sp}$—interneuron’s spike times.

Astrocytes play a crucial role in maintaining the homeostasis of ions and neurotransmitters at the synapse [33]. Each astrocyte extends several primary processes (defined as branches), which split into increasingly smaller processes, eventually ending in peripheral processes with sheet-like structures known as leaflets. These leaflets lack organelles but have various transporters dwelling in their membrane [34,35]. Astrocytic leaflets connect with synapses thus forming a tripartite synapse and maintaining synaptic microenvironment by regulating ionic and neurotransmitters concentrations.

In this study, the perisynaptic cradle (PsC) refers to the astrocytic compartment that surrounds synapses and regulates ion and neurotransmitter concentrations in the synaptic cleft. The PsC includes an astrocytic leaflet as well.

The ion and neurotransmitter currents within the PsC are calculated using Equations (16)–(20):

(16) $$I_{Na,ast}=-3I_{EAAT,ast}+3I_{NCX,ast}+3I_{NKA,ast}+2I_{GAT,ast}+I_{NaL,ast},$$

(17) $$I_{K,ast}=I_{EAAT,ast}-2I_{NKA,ast}+I_{Kir,ast}+I_{KL,ast},$$

(18) $$I_{Ca,ast}=-I_{NCX,ast}+I_{CaL,ast},$$

(19) $$I_{GABA,ast}=I_{GAT,ast}+I_{GABAL,ast},$$

(20) $$I_{Glu,ast}=I_{EAAT,ast}+I_{GluL,ast}.$$

where $I_{EAAT}$, $I_{NCX}$, $I_{Kir}$ and $I_{GAT}$ denote EAAT-2, NCX, Kir4.1 and GAT-3 generated currents, respectively.

Ion diffusion within the leaflet is also considered, as described by Equation (21):

(21) $$I_{pf_{X}}=K_{pf}\frac{-E_{X_{ast}}}{l_{lf}}\exp\left(-\varphi +\frac{Q}{k_{B}T}\sqrt{\frac{Q\left(-E_{X_{ast}}\right)}{l_{lf}\pi \varepsilon }}\right),\;\;X\in \left\{Na,K,Ca\right\}.$$

Here, $I_{{pf}_{X}}$ denotes leaflet diffusion current; $E_{X_{ast}}$ represents equilibrium potentials for ions relative to the intracellular space of the astrocyte; $l_{lf}$ is the leaflet length; $K_{pf}$, φ, ε, Q, $k_{B}$ and T denote the Poole–Frankel channel constant, well activation energy, dynamic permittivity, elementary charge, Boltzmann’s constant and absolute temperature, respectively.

The dynamics of the extracellular space (ECS) are determined by considering ion and neurotransmitter currents from all other compartments, as well as ion diffusion within the cleft, as described by Equations (22) and (23):

(22) $$I_{X_{ecs}}=I_{X_{dif_{ecs}}}-I_{X_{psc}}-I_{X_{post}}-I_{X_{pre}},\;\;X\in \left\{Na,K,Ca\right\},$$

(23) $$I_{X_{ecs}}=-I_{X_{psc}}+X_{rel},\;\;X\in \left\{GABA,Glu\right\},$$

where $I_{X_{{dif}_{ecs}}}$ represents ECS diffusion current and $X_{rel}$ denotes the concentration of neurotransmitters released by the presynaptic neuron and interneuron.

ECS diffusion is calculated relative to the global extracellular space (GECS), which refers to the extensive extracellular region surrounding neurons and glial cells, as described by Equation (24):

(24) $$I_{X_{dif_{ecs}}}=g_{ecs}\lambda E_{X_{ecs}}SA_{ecs_{dif}},$$

where $E_{X_{ecs}}$ denotes equilibrium potentials for ions relative to the GESC; ${SA}_{{ecs}_{dif}}$ is the leaflet cross-sectional area; λ and $g_{ecs}$ denote conductance scaling factor and maximal diffusion conductance, respectively. It is assumed that the GESC has a much larger volume than the ECS; therefore, the concentrations of all ions in the GECS are assumed to remain effectively constant.

### 2.5. Numerical Simulation Results

#### 2.5.1. Preliminary Information

The results of the numerical simulations for the modular model of neuronal activity are presented for the entire simulation period of 100 s. The presynaptic and postsynaptic neurons are stimulated by an externally applied pulsed current for 50 s, starting at the 10 s. Inset graphs display the last second of stimulation to provide a more detailed view of the dynamics of currents, concentrations, and membrane potentials. Positive values of transmembrane currents indicate exiting the cellular compartment (outward flux), while negative values represent entering the cellular compartment (inward flux).

#### 2.5.2. Neuronal Activity

**Figure 3:**
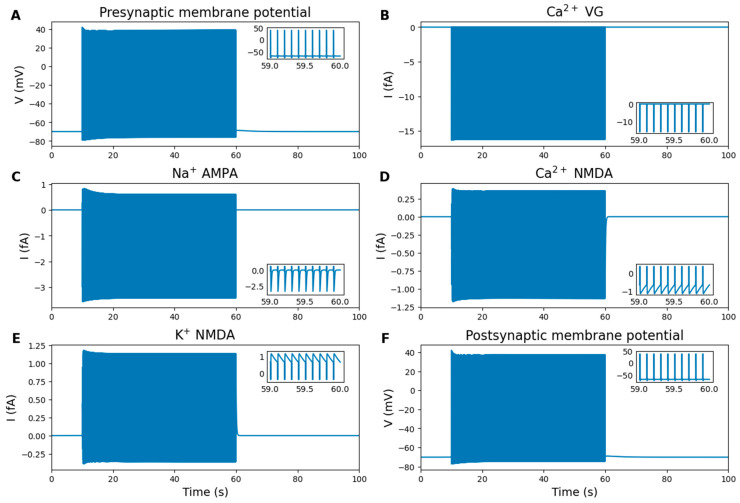
Numerical simulation results: neuronal activity. Inset graphs display the last second of stimulation. (**A**) Pre membrane potential. (**B**) Pre voltage-gated Ca^2+^ channels current. (**C**) Post AMPA-Rs Na+ current. (**D**) Post Ca^2+^ NMDA-Rs current. (**E**) Post K^+^ NMDA-Rs current. (**F**) Post membrane potential. Presynaptic depolarization (**A**) activates voltage-gated Ca^2+^ channels (**B**), triggering neurotransmitter release. Postsynaptic AMPA and NMDA receptors mediate Na^+^ (**C**) and Ca^2+^ (**D**) influx, while NMDA receptors also allow K^+^ efflux (**E**), leading to postsynaptic depolarization (**F**).

The presynaptic neuron (Pre) is stimulated with a pulsed depolarizing current of 10 µA/cm^2^, at a frequency of 10 Hz, and a pulse width of 3 ms. This frequency was chosen to provide stable synaptic activation and is commonly used in computational modeling studies of tripartite synapse dynamics [11,25,36]. The stimulation causes membrane depolarization (Figure 3A), which triggers the opening of voltage-gated Na^+^, K^+^ and Ca^2+^ channels, leading the neuron to generate action potentials. The action potential of the Pre has a magnitude of approximately 110 mV, with ~100 mV depolarization and ~10 mV afterhyperpolarization.

Glutamate and GABA are the brain’s primary excitatory and inhibitory neurotransmitters, respectively [37]. During each action potential, Ca^2+^ enters the Pre through voltage-gated Ca^2+^ channels (Figure 3B), leading to glutamate release into the synaptic cleft. Glutamate then activates AMPA-Rs and NMDA-Rs on the postsynaptic neuron (Post). Activation of AMPA-Rs allows Na^+^ to flow into the Post (Figure 3C), while NMDA-Rs permit both Na^+^ and Ca^2+^ to enter the cell (Figure 3D), with K^+^ efflux into the synaptic cleft (Figure 3E). The activity of glutamate receptors depolarizes the Post membrane, triggers the opening of voltage-gated ion channels, and initiates action potentials (Figure 3F). In contrast, GABA serves as an inhibitory neurotransmitter by binding to GABA_A_-Rs and GABA_B_-Rs, thereby retarding synaptic transmission. Under normal conditions, GABA levels in the ECS remain low, providing a mild but consistent inhibitory effect on nearby neurons. However, external sources of GABA, such as interneurons, can significantly increase its concentration, leading to a substantial decrease in synaptic activity (as shown in Section 2.5.5).

To remove the magnesium block of NMDA-Rs, the Post is stimulated with a weak depolarizing current simultaneously with glutamate release from the Pre. This depolarization alone is insufficient to induce postsynaptic firing and does not substitute synaptic excitation mediated by AMPA-Rs activation. Notably, the slight reduction in action potential amplitudes in both the Pre and Post after peaking at the onset of stimulation may reflect the mechanism of short-term depression [38].

#### 2.5.3. Astrocytic Regulation

**Figure 4:**
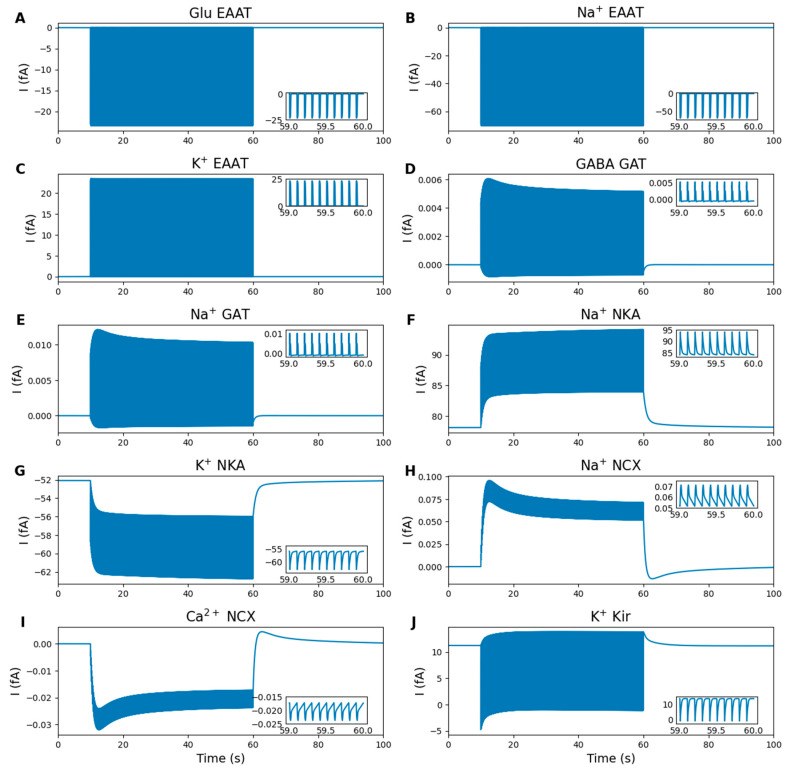
Numerical simulation results: astrocytic transmembrane currents. Inset graphs display the last second of stimulation. (**A**–**C**) Glutamate, Na^+^ and K^+^ EAAT-2 generated currents. (**D**,**E**) GABA and Na^+^ GAT-3 generated currents. (**F**,**G**) Na^+^ and K^+^ NKA generated currents. (**H**,**I**) Na^+^ and Ca^2+^ NCX generated currents. (**J**) K^+^ current through the Kir4.1 channels. Astrocytes regulate neurotransmitter and ion homeostasis via EAAT-2, GAT-3, NKA, NCX, and Kir4.1. Glutamate uptake via EAAT-2 (**A**) is accompanied by Na^+^ influx (**B**) and K^+^ efflux (**C**), while Na^+^-dependent GAT-3 activation releases GABA (**D**) and Na^+^ (**E**), contributing to synaptic inhibition. NKA (**F**,**G**), NCX (**H**,**I**) and Kir4.1 (**J**) help maintain ionic homeostasis.

Figure 4 shows the ion and neurotransmitter currents through the main astrocytic pathways: EAAT-2, NCX, NKA, Kir4.1 and GAT-3. Glutamate released into the ECS during Pre stimulation is taken by the astrocytic EAAT-2 (Figure 4A), leading to an influx of Na^+^ (Figure 4B) and an efflux of K^+^ (Figure 4C). According to published data [25], the Na^+^ influx through EAAT-2 is sufficient to activate the GAT-3, which releases GABA (Figure 4D) and Na^+^ (Figure 4E) into the synapse, contributing to neuronal inhibition. The maintenance of ionic homeostasis also involves NKA (Figure 4F,G), NCX (Figure 4H,I) and Kir4.1 (Figure 4J). The current through NCX is significantly lower than that through EAAT-2, consistent with experimental observations [39]. The slow release of GABA by the GAT-3 contributes to long-lasting tonic inhibition of nearby neurons, sustaining a low yet steady GABA level in the synaptic cleft [26,40,41]. This contrasts with the transient, or phasic, inhibition typically caused by exocytosis [41].

#### 2.5.4. Ion and Neurotransmitter Concentrations

**Figure 5:**
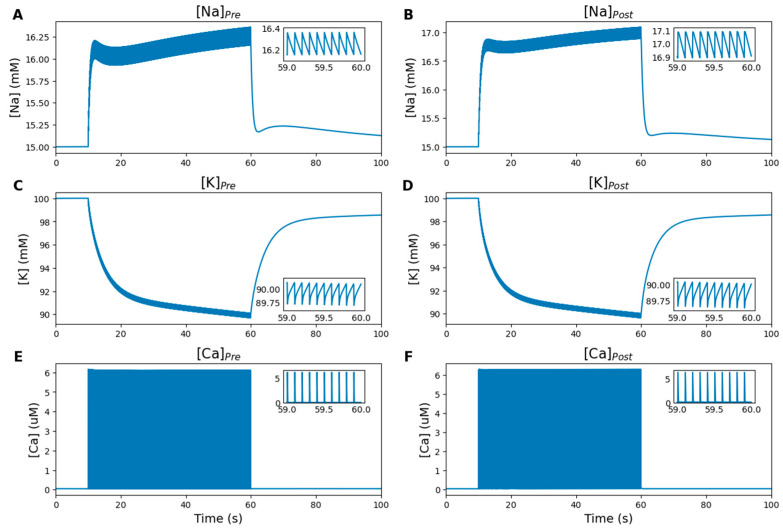
Numerical simulation results: ionic concentrations in the Pre and Post. Inset graphs display the last second of stimulation. (**A**,**C**,**E**) Concentrations of Na^+^, K^+^ and Ca^2+^ in the Pre. (**B**,**D**,**F**) Corresponding concentrations in the Post. During stimulation, Na^+^ and Ca^2+^ enter the neurons (**A**,**B**,**E**,**F**) while K^+^ flows out (**C**,**D**), driven by their electrochemical gradients. After stimulation ends, NKA and PMCA restore ionic homeostasis.

Figure 5 illustrates the dynamics of ionic concentrations in the presynaptic and postsynaptic neurons. Resting ion and neurotransmitter concentrations were set according to experimentally derived physiological data reported in the literature [42,43]. Membrane depolarization causes the opening of voltage-gated ion channels, allowing ion currents to flow according to their concentration gradients, resulting in Na^+^ and Ca^2+^ influxes and K^+^ efflux (Figure 5A–F). Once the stimulation ends, the ion concentrations quickly return to their equilibrium levels due to the activity of NKA and PMCA.

**Figure 6:**
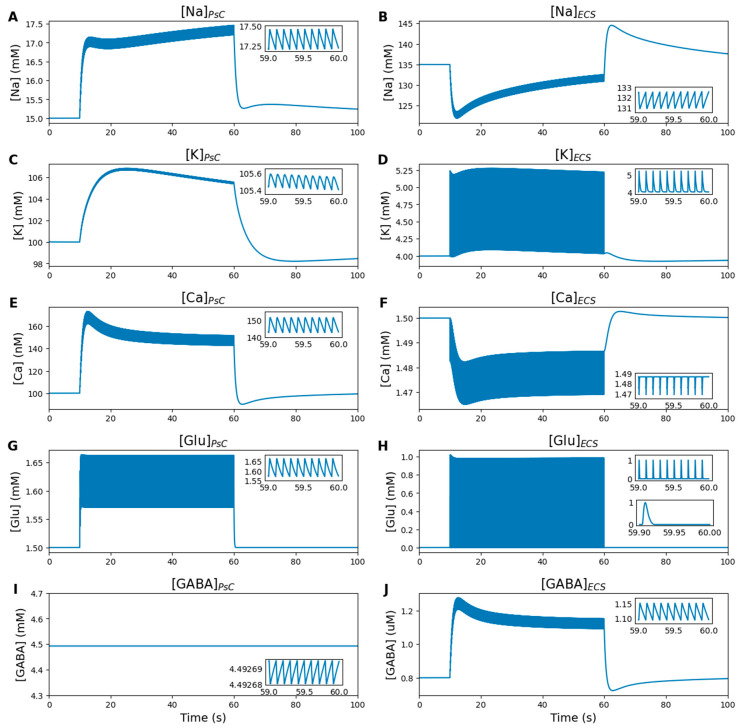
Numerical simulation results: ion and neurotransmitter concentrations in the PsC and ECS. Inset graphs display the last second of stimulation. (**A**,**C**,**E**,**G**,**I**) Concentrations of Na^+^, K^+^, Ca^2+^, glutamate and GABA in the PsC. (**B**,**D**,**F**,**H**,**J**) Corresponding concentrations in the ECS. For glutamate in the ECS (**H**), an additional inset shows the last 100 ms of stimulation, capturing a single peak in concentration. Synaptic activity drives transient increases in extracellular glutamate (**H**) and GABA (**J**), which are cleared by astrocytic transporters (**G**,**I**). Ionic homeostasis is maintained by coordinated ion transport, including Na^+^ influx into the PsC via EAAT-2 (**A**), K^+^ shuttling between the ECS and PsC (**C**,**D**), and Na^+^/Ca^2+^ exchange through NCX (**A**,**B**,**E**,**F**).

The dynamics of ion and neurotransmitter concentrations in the PsC and ECS are shown in Figure 6. Synaptic activity alters synaptic concentrations, with astrocytes actively regulating synaptic homeostasis.

When presynaptic vesicles release their contents, glutamate concentrations in the synaptic cleft quickly peak around 1 mM (Figure 6H). This glutamate is rapidly cleared from the synapse by the astrocytic EAAT-2 and subsequently decays (Figure 6G). The clearance time for glutamate from the synaptic space is approximately 30 ms (Figure 6H, lower inset), consistent with experimental observations [44]. During each EAAT-2 transport cycle, Na^+^ is transported into the PsC (Figure 6A), while K^+^ is moved out to the ECS (Figure 6D). Astrocytic NKA prevents excessive K^+^ accumulation in the ECS, which could result from NMDA-Rs, EAAT-2 and Kir4.1 (Figure 6C), and facilitates Na^+^ efflux from the astrocyte (Figure 6B). Changes in extracellular K^+^ concentration remain within the acceptable increase range of several millimolars during periods of intense neuronal activity [45]. The activity of NCX is highly dependent on the intra- and extracellular Na^+^ concentrations, so the dynamics of Ca^2+^ concentrations are mainly determined by Na^+^ levels in the PsC and ECS (Figure 6E,F).

In the absence of inhibitory input from interneurons, extracellular GABA concentration is maintained at a low but constant level (around 1 µM, Figure 6J). The weak current of GABA is not sufficient to significantly change its concentration in the PsC (Figure 6I). The results of the simulation under conditions of interneuron inhibition are presented in the following section.

After stimulation ends, all concentrations return to their equilibrium levels. Na^+^ and Ca^2+^ concentrations quickly recover due to the activity of NKA and NCX, which pump these ions out of the PsC. K^+^ concentration recovery is slower, as K^+^ continues to shuttle between the ECS and PsC through NKA, Kir4.1 and leak channels.

#### 2.5.5. Interneuron Inhibition

**Figure 7:**
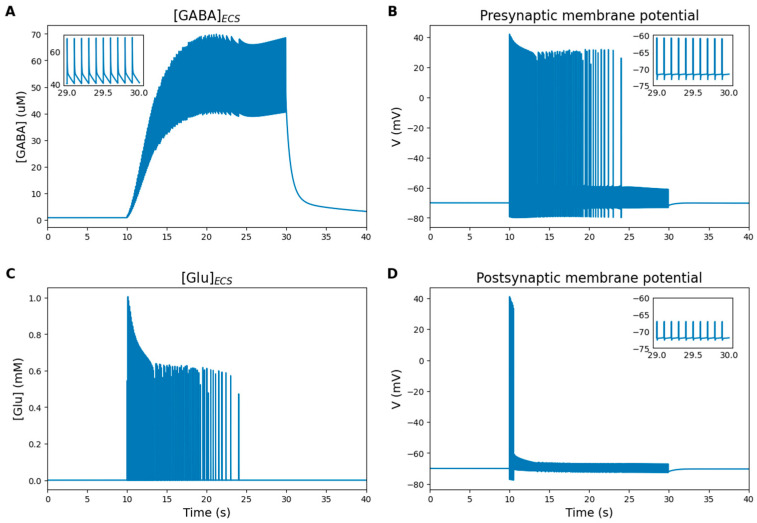
Numerical simulation results under conditions of interneuron inhibition. In this simulation, the Pre and interneuron were stimulated for 20 s, with the total simulation time being 40 s. Inset graphs display the last second of stimulation. (**A**) GABA concentration in the ECS. (**B**) Pre membrane potential. (**C**) Glutamate concentration in the ECS. (**D**) Post membrane potential. Interneuron activation leads to a rapid GABA increase in the ECS (**A**), reducing glutamate release (**C**) and suppressing synaptic transmission. This inhibition lowers the frequency and amplitude of Pre and Post membrane oscillations (**B**,**D**), eventually preventing action potential generation.

At rest, GABA concentration in the synaptic cleft remains low (around 0.8 µM [46]). Interneurons are able to provide inhibition to neuronal networks and dictate the temporal pattern of activity of pyramidal neurons [46]. Stimulation of the interneuron leads to the opening of voltage-gated channels and the subsequent exocytosis of synaptic vesicles containing GABA. This results in a rapid increase in GABA concentration in the synaptic cleft, reaching levels far above the baseline (tens of µM, Figure 7A). The large fluctuations in GABA concentration are driven by the active uptake mechanism of the GAT-3 transporter.

In the ECS, GABA binds to GABA_A_-Rs and GABA_B_-Rs on neuronal surfaces, generating inhibitory currents and reducing the likelihood of glutamate release. These factors lead to a decrease in the frequency and amplitude of membrane potential oscillations (Figure 7B,D) and a reduction in glutamate synaptic concentration (Figure 7C). Gradually, the membranes of both neurons stop depolarizing beyond the threshold, halting action potential generation and thereby stopping synaptic transmission.

The Pre remained in an excited state longer than the Post because it is affected by an external stimulus with high amplitude, whereas the only source of excitation of the Post is the activation of glutamate receptors.

It should be noted that the accumulation of excessive GABA concentration in the synapse can also be caused by a number of other factors, including the inhibition of GABA transporter GAT-1 and GABA aminotransferase GABA-T, which are the basis for the mechanisms of action of some antiepileptic drugs (e.g., valproic acid [47]). While these components are not considered in our model, we assume that the increase in GABA levels through interneuron release has a similar impact on system dynamics, as the primary outcome of targeting GAT-1 and GABA-T is the elevation of GABA concentration in the synaptic cleft.

#### 2.5.6. Simulation of Drug Effect

One of the most common targets of AEDs is voltage-gated Na^+^ channels [48]. Inhibitors of these channels include drugs like carbamazepine, lamotrigine, phenytoin, lacosamide, and others [23]. These drugs bind predominantly to depolarized Na^+^ channels, shifting them into a non-conducting state similar to inactivation but with slower recovery. Inhibitory effect occurs during prolonged or repetitive neuronal depolarization, which is crucial during epileptic seizures [48]. The drug concentration is calibrated to prevent pathological activity without completely blocking neuronal function. This approach ensures a balance between therapeutic effectiveness and the preservation of normal neuronal activity.

To model the effect of Na^+^ channel inhibitors, one can reduce the conductance of these channels and/or adjust the parameters governing their activation and inactivation kinetics. As an example, we will gradually decrease the maximal conductance of voltage-gated Na^+^ channels starting from the point of stimulus application, thereby simulating the selective enhancement of their slow inactivation.

**Figure 8:**
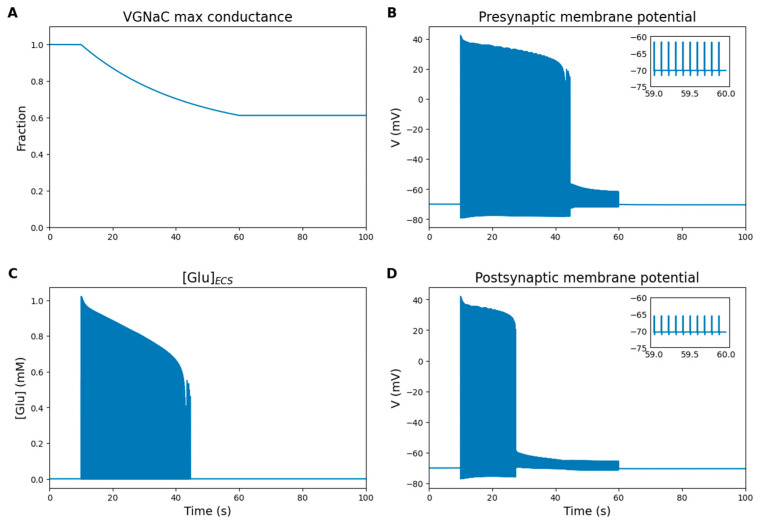
Numerical simulation results under conditions of voltage-gated Na^+^ channels inhibition. Inset graphs display the last second of stimulation. (**A**) Fraction of maximal voltage-gated Na^+^ channels conductance. (**B**) Pre membrane potential. (**C**) Glutamate concentration in the ECS. (**D**) Post membrane potential. Reduction in maximal Na^+^ channel conductance decreases action potential amplitude. When the reduction exceeds ~30%, synaptic transmission is completely inhibited.

Figure 8 shows the simulation results under conditions of voltage-gated Na^+^ channels inhibition. When the maximal conductance is reduced by less than 20% (Figure 8A), the Na^+^ channels still provide enough current to generate action potentials (Figure 8B). With a reduction of more than 20% (Figure 8A), the Pre continues to generate action potentials in the presence of a strong external stimulus (Figure 8B), but the glutamate concentration released (Figure 8C) is insufficient to excite the Post (Figure 8D). Reduction of more than 30% (Figure 8A) leads to complete inhibition of synaptic transmission (Figure 8D).

A study on differentiated NG108-15 cells, which are frequently used in in vitro studies instead of primary-cultured neurons, demonstrated that a decrease in voltage-gated Na^+^ channel availability suppresses action potential generation, while a sufficient level of Na^+^ conductance is required to sustain neuronal excitability [49]. In our model, a gradual reduction in maximal Na^+^ channel conductance leads to a progressive decrease in action potential amplitude. Once a critical threshold (~30% reduction) is reached, action potential generation ceases entirely, as the membrane potential no longer surpasses the firing threshold. This result supports the idea that Na^+^ channel inhibition can significantly impact neuronal firing and synaptic function.

Thus, the model is capable of simulating the basic effects of pharmacological treatments. Given the complexity of AED mechanisms and the limited availability of experimental data, a thorough investigation requires extensive parameter optimization and validation. This aspect will be the focus of our future work. Meanwhile, the developed modular model serves as a valuable standalone framework for simulating neuronal activity while accounting for the dynamics of the main molecular targets of AEDs.

## 3. Discussion

Synaptic transmission is a widely researched area in both experimental and computational research. In this study, we developed a mathematical model of neuronal activity that incorporates the dynamics of main molecular targets of AEDs, the role of astrocytic transporters in regulating ion and neurotransmitter concentrations, and the impact of interneuron inhibition.

The constructed model describes synaptic activity by calculating changes in ion and neurotransmitter concentrations which are consistent with experimental data. A key advantage of the model is that it includes the dynamics of main molecular targets of AEDs, which opens up opportunities for modeling drug effects on synaptic transmission, ranging from changes in ion and neurotransmitter concentrations to broader impacts on neuronal networks. Specifically, the model explicitly accounts for voltage-gated Na^+^, K^+^ and Ca^2+^ channels, AMPA, NMDA, GABA_A_ and GABA_B_ receptors, as well as key astrocytic transporters, including the glutamate transporter EAAT-2 and the GABA transporter GAT-3.

For model construction, we selected biophysically detailed nanoscopic-level neuronal activity models that incorporate the dynamics of the main molecular targets of AEDs and were best suited for integration into a unified framework. These selected models are listed in Table 1.

Beyond the models integrated into the modular model, we acknowledge the existence of many other noteworthy neuronal activity models. However, they were not directly suitable for integration due to specific limitations. For example, some models focus exclusively on the detailed dynamics of a single neuron [13,50,51], or describe astrocytic regulation of presynaptic activity [52,53,54] without considering synaptic transmission, which is crucial in the context of epilepsy. Additionally, some models adopt a population-based approach, averaging neuronal ensemble activity instead of capturing the dynamics of individual neurons [55,56]. While these models provide valuable insights into specific aspects of neuronal dynamics, our approach aims for a detailed representation of synaptic transmission at the level of single neurons. Finally, several biophysical models of the tripartite synapse have been proposed [11,57,58,59], which could have been used as a foundation for our modular model. However, none of them simultaneously account for all the main molecular targets of AEDs considered in our study. We selected [11] as the most suitable basis because it already incorporated many of the key processes relevant to our objectives while omitting unrelated mechanisms. This choice minimized the need for additional modifications to incorporate the main molecular targets of AEDs while preserving biological accuracy.

The current formulation of the model incorporates a relatively simple Hodgkin-Huxley formalism to describe the dynamics of voltage-gated ion channels. This approach provides a biologically meaningful representation of action potential generation and ionic currents, which is sufficient for capturing the dynamics of the main molecular targets of antiepileptic drugs. However, this approach has inherent limitations in reproducing finer effects of pharmacological interventions, such as representation of drug-binding and unbinding kinetics, voltage-dependent channel affinity, or plasma concentration-time profiles. The goal of the present study is not to reproduce detailed experimental or clinical pharmacokinetic/pharmacodynamic relationships, but to establish a biophysically detailed model that explicitly accounts for the dynamics of all the main molecular targets of AEDs, including ion channels, synaptic receptors, and astrocytic transporters. This foundation is necessary to enable systematic extension and refinement of the model in future studies.

Similarly, the model employs simplified representations of GABA_B_ and mGlu receptor activation, which limits its ability to capture the full diversity of downstream signaling pathways associated with these receptors. Clarification and explicit modeling of the corresponding molecular cascades constitute an additional direction for further development of the model.

Also, at the present stage, the model does not explicitly reproduce epileptiform discharges or ictal–interictal transitions; its primary purpose is to capture the dynamics of the main molecular targets of antiepileptic drugs under physiologically relevant neuronal activity. In the future, we plan to add a pathological excitation pathway to the model with the aim of simulating seizures and exploring epilepsy drug therapy. The extended model will then be integrated into our previously published multilevel model of epileptic seizures [60] to study drug therapy effects across different levels of brain organization.

## 4. Methods

### 4.1. Modeling Software

The construction and numerical calculations of the model were carried out on the BioUML platform (https://sirius-web.org/bioumlweb/, version 2025.2, accessed on 1 January 2026), an open-source Java-based integrated environment designed for modeling complex biological systems and analyzing biomedical data [61,62]. In this section, we provide a brief overview of the specific functionality of the BioUML platform that was used in the creation of the model.

### 4.2. Visual Modeling

Graphical representation simplifies the development of mathematical models for biological systems. In the visual modeling approach, processes and systems are depicted as diagrams, which serve as the basis for numerical simulations.

The model was constructed on the BioUML platform using visual modeling as follows. Initially, the biological system is represented as a diagram, from which BioUML generates Java code that describes the corresponding system of differential equations. This code is then compiled and executed by the numerical solvers integrated into BioUML to simulate the system’s dynamics.

### 4.3. Modular Modeling

The model was developed using a modular approach implemented in the BioUML platform, which allows any complex system to be broken down into elementary components called modules (also referred to as submodels or blocks). Each module can be created and configured independently, using its own formalism and level of detail. The interaction between modules is defined by the connections between their internal variables. These connections represent signal transmission pathways between the corresponding blocks. The graphical elements of the modular approach used in model construction are presented in Table 2.

Constructing a modular model involves defining submodels, specifying their input and output variables using ports, and connecting these ports through appropriate connections. Each module can have multiple connections with other modules. Additionally, in order to simplify the visual representation of the model, a bus element was used in the modular diagram to allow remote connections between modules.

### 4.4. Numerical Solution

One unit of model time corresponds to one second of real time. All simulation results presented in this work were performed using a forward Euler numerical integration scheme with a fixed time step $dt=10^{-6}$ (1 μs), with the simulation time set to 100 s.

## 5. Conclusions

Within this paper, the modular computer model of neuronal activity has been presented. It captures the dynamics of the main molecular targets of antiepileptic drugs, including voltage-gated Na^+^, K^+^ and Ca^2+^ channels, as well as AMPA, NMDA and GABA receptors.

The model simulates the signal transmission from the presynaptic to the postsynaptic neuron across the synaptic cleft, where the dynamics of ion and neurotransmitter concentrations are actively regulated by astrocytes through various channels and transporters, including the GABA transporter GAT-3, which is particularly important in modeling excessive synaptic activity. Additionally, the system incorporates an interneuron that can inhibit synaptic transmission by releasing GABA.

One of a key advantage of the model is its ability to simulate the effects of antiepileptic drugs on their molecular targets. As an example, the results of simulation under conditions of voltage-gated Na^+^ channels inhibition were presented.

Comparative analysis of the model’s numerical results with data from the literature revealed realistic reproduction of ion and neurotransmitter concentration dynamics, as well as currents through astrocytic channels, transporters and exchangers. The developed model is well-suited for use in the study of epilepsy drug therapy.

## Figures and Tables

**Table 1:** Neuronal activity models integrated into the modular model.

Model	Crucial Components
Toman et al., 2023 [11]	VGKC, VGNaC, VGCaC, AMPAR, NMDAR, Glu
Flanagan et al., 2021 [25]	VGKC, VGNaC, AMPAR, NMDAR, GABA_A_R, mGluR, GAT-3, Glu, GABA
Borjkhani et al., 2018 [12]	VGKC, VGNaC, VGCaC, AMPAR, NMDAR, Glu, GABA
Li et al., 2020 [28]	AMPAR, NMDAR, GABA_B_R, mGluR, Glu, GABA

Abbreviations: 1. VGKC, VGNaC, VGCaC—voltage-gated Na^+^, K^+^ and Ca^2+^ channels. 2. AMPAR, NMDAR, GABA_A_R, GABA_B_R, mGluR—AMPA, NMDA, GABA_A_, GABA_B_ and metabotropic glutamate receptors. 3. GAT-3—astrocytic GABA-transporter 3. 4. Glu, GABA—neurotransmitters glutamate and GABA.

**Table 2:** Graphical elements of the modular approach used in model construction.

Graphical Notation	Name	Description
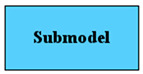	Submodel	Module that can contain either a model of a subsystem or an entire modular model. Input and output variables are defined by ports
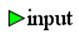	Input port	Port that defines the input variable of the submodel
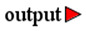	Output port	Port that defines the input variable of the submodel
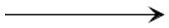	Directed connection	Connection corresponds to a variable value calculated in one module and then passed to another module
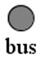	Bus	Variable in the modular model. Multiple buses may correspond to a single variable

## Data Availability

The implementation of the model is available in the web version of the BioUML platform and is stored in the GitLab repository at: https://gitlab.sirius-web.org/brain/neuronal-activity-model-with-aed-targets, accessed on 1 January 2026.

## Appendix A. Presynaptic Neuron Compartment

**Table A1:** Equations of the presynaptic neuron compartment.

Equation	Description	Source
$C\frac{dV}{dt}=I_{app}-\left(I_{Na}+I_{K}+I_{Ca}+I_{L}+I_{GABAA}\right)$	Change in membrane potential	Adapted from [11,25]
$I_{Na_{vg}}=g_{Na}m^{3}h\left(V-E_{Na}\right)$	Voltage-gated Na^+^ channels current	[11]
$I_{K_{vg}}=g_{K}n^{4}\left(V-E_{K}\right)$	Voltage-gated K^+^ channels current	[11]
$I_{Ca_{vg}}=g_{Ca}m_{Ca}^{3}\left(V-E_{Ca}\right)$	Voltage-gated Ca^2+^ channels current	[11]
$I_{L}=g_{L}\left(V-E_{L}\right)$	Background leak channels current	[11]
$\frac{dw}{dt}=\alpha_{w}\left(1-w\right)-\beta_{w}w$, where $w\in \left\{m,h,n\right\}$	Changes in gating variables for Na^+^ and K^+^ in a Hodgkin-Huxley formalism	[11]
$\frac{dm_{Ca}}{dt}=\frac{m_{Ca_{\inf}}-m_{Ca}}{\tau_{m_{Ca}}}$	Change in gating variable for Ca^2+^	[11]
$\alpha_{m}=\frac{2.5-0.1\left(V-V_{rest}\right)}{\exp\left(2.5-0.1\left(V-V_{rest}\right)\right)-1}$	Voltage-gated Na^+^ channel activation forward rate	[11]
$\beta_{m}=4\exp\left(\frac{V_{rest}-V}{18}\right)$	Voltage-gated Na^+^ channel activation backward rate	[11]
$\alpha_{h}=0.07\exp\left(\frac{V_{rest}-V}{20}\right)$	Voltage-gated Na^+^ channel inactivation forward rate	[11]
$\beta_{h}=\frac{1}{1+\exp\left(3-0.1\left(V-V_{rest}\right)\right)}$	Voltage-gated Na^+^ channel inactivation backward rate	[11]
$\alpha_{n}=\frac{0.1-0.01\left(V-V_{rest}\right)}{\exp\left(1-0.1\left(V-V_{rest}\right)\right)-1}$	Voltage-gated K^+^ channel activation forward rate	[11]
$\beta_{n}=0.125\exp\left(\frac{V_{rest}-V}{80}\right)$	Voltage-gated K^+^ channel activation backward rate	[11]
$m_{Ca_{\inf}}=\frac{1}{1+\exp\left(-\frac{V+24.758}{8.429}\right)}$	Voltage-gated Ca^2+^ channel equilibrium activation rate	[11]
$\tau_{m_{Ca}}=\left\{\begin{array}{l} 0.2702+1.1622\exp\left(\frac{-{\left(V+22.098\right)}^{2}}{164.19}\right),\mathrm{if}\;V\geq -40\;,\\ 0.6923\exp\left(\frac{V-4.7}{1089.372}\right),\;\;\mathrm{otherwise}. \end{array}\right.$	Voltage-gated Ca^2+^ channel activation time	[11]
$I_{Na}=I_{Na_{vg}}\cdot conv+3I_{NKA}+I_{NaL}$	Na^+^ membrane current density	Adapted from [11]
$I_{K}=I_{K_{vg}}\cdot conv-2I_{NKA}+I_{KL}$	K^+^ membrane current density	Adapted from [11]
$I_{Ca}=I_{Ca_{vg}}\cdot conv+I_{PMCA}+I_{CaL}$	Ca^2+^ membrane current density	Adapted from [11]
$I_{X_{pre}}=I_{X}SA_{pre}$, where $X\in \left\{Na,K,Ca\right\}$	Na^+^, K^+^ and Ca^2+^ membrane currents	[11]
$I_{GABAA}=g_{GABAA}r_{GABAA}\left(V-E_{GABAA}\right)$	GABA_A_-R current	[25]
$\frac{dr_{GABAA}}{dt}=\alpha_{GABAA}GABA_{ecs}\left(1-r_{GABAA}\right)-\beta_{GABAA}r_{GABAA}$	Change in fraction of activated GABA_A_-Rs	[25]
$I_{NKA}=I_{NKA_{\max}}F\frac{Na^{1.5}}{Na^{1.5}+{K_{dNa}}^{1.5}}\frac{K_{ecs}}{K_{ecs}+K_{dK}}$	NKA current	[11]
$I_{PMCA}=I_{PMCA_{\max}}F\frac{Ca}{K_{PMCA}+Ca}$	PMCA current	[11]
$I_{XL}=g_{XL}\left(V-E_{X}\right)$, where $X\in \left\{Na,K,Ca\right\}$	Na^+^, K^+^ and Ca^2+^ leak currents	[11]
$E_{X}=\frac{RT}{z_{X}F}\ln\left(\frac{X_{ecs}}{X}\right)$, where $X\in \left\{Na,K,Ca\right\}$	Equilibrium potentials for Na^+^, K^+^ and Ca^2+^	[11]
$J_{Na_{pre}}=-\frac{I_{Na_{pre}}}{z_{Na}F\cdot Vol_{pre}}$, where $X\in \left\{Na,K,Ca\right\}$	Na^+^, K^+^ and Ca^2+^ membrane fluxes	[11]
$\frac{dX}{dt}=J_{X_{pre}}$, where $X\in \left\{Na,K,Ca\right\}$	Changes in Na^+^, K^+^ and Ca^2+^ concentrations	[11]
$\frac{dQ}{dt}=\left(k_{1}\left(Ca-Ca_{rest}\right)\left(1-Q\right)-k_{-1}Q-nb\cdot k_{2}Q^{nb}\right)$	Change in the concentration of activated synaptic vesicles	[11]
$\frac{dRR}{dt}=\left(k_{2}Q^{nb}-k_{3}RR\right)$	Change in the rate of glutamate release	[11]
$Glu_{rel}=U\cdot T_{\max}\cdot RR$	Concentration of glutamate released per second	[11]
$U=U_{0}+\left(1-U_{0}\right)r_{mGluR}-U_{0}r_{GABAB}$	Probability of glutamate release	[28]
$\frac{dr_{mGluR}}{dt}=\alpha_{mGluR}Glu_{ecs}\left(1-r_{mGluR}\right)-\beta_{mGluR}r_{mGluR}$	Change in fraction of activated mGlu-Rs	[28]
$\frac{dr_{GABAB}}{dt}=\alpha_{GABAB}GABA_{ecs}\left(1-r_{GABAB}\right)-\beta_{GABAB}r_{GABAB}$	Change in fraction of activated GABA_B_-Rs	[28]

**Table A2:** Parameter values of the presynaptic neuron compartment.

Parameter	Description	Value	Source
F	Faraday’s constant	96,485.33 C·mol^−1^	–
R	Ideal gas constant	8.3145 J·mol^−1^·K^−1^	–
T	Absolute temperature	310 K	–
C	Membrane capacitance	1 µF·cm^−2^	[11]
$Vol_{pre}$	Pre volume	0.014314 fl	[11]
$SA_{pre}$	Pre surface area	0.21206 µm^2^	[11]
$V_{rest}$	Pre resting potential	−70 mV	[11]
$g_{Na}$	Maximal voltage-gated Na^+^ channels conductance	120 mS·cm^−2^	[11]
$g_{K}$	Maximal voltage-gated K^+^ channels conductance	36 mS·cm^−2^	[11]
$g_{Ca}$	Maximal voltage-gated Ca^2+^ channels conductance	0.1 mS·cm^−2^	[11]
$g_{L}$	Maximal leak channels conductance	0.3 mS·cm^−2^	[11]
$g_{GABAA}$	Maximal GABA_A_-Rs conductance	0.05 mS·cm^−2^	[25]
$E_{GABAA}$	Equilibrium potential for GABA_A_-Rs	−85 mV	[25]
$\alpha_{GABAA}$	GABA_A_-R activation rate	500 × 10^3^ M^−1^ s^−1^	[25]
$\beta_{GABAA}$	GABA_A_-R inactivation rate	720 s^−1^	[25]
$I_{NKA_{\max}}$	Maximal NKA velocity	1.12 × 10^−6^ mol·m^−2^ s^−1^	[11]
$K_{dNa}$	NKA Na^+^ affinity	0.01 M	[11]
$K_{dK}$	NKA K^+^ affinity	0.6 × 10^−3^ M	[11]
$I_{PMCA_{\max}}$	Maximal PMCA velocity	0.2 × 10^−6^ mol·m^−2^ s^−1^	[11]
$K_{PMCA}$	PMCA Ca^2+^ affinity	0.2 × 10^−6^ M	[11]
$g_{NaL}$	Na^+^ leak conductance	${\left.-\frac{I_{Na}\cdot conv+3I_{NKA}}{V-E_{Na}}\right|}_{t=0}$	Calculated for model stability
$g_{KL}$	K^+^ leak conductance	${\left.-\frac{I_{K}\cdot conv-2I_{NKA}}{V-E_{K}}\right|}_{t=0}$	Calculated for model stability
$g_{CaL}$	Ca^2+^ leak conductance	${\left.-\frac{I_{Ca}\cdot conv+I_{PMCA}}{V-E_{Ca}}\right|}_{t=0}$	Calculated for model stability
$Ca_{rest}$	Ca^2+^ resting concentration	50 × 10^−9^ M	[11]
$k_{1}$	Vesicle activation rate	0.5 ms^−1^	[11]
$k_{-1}$	Vesicle inactivation rate	1 ms^−1^	[11]
$k_{2}$	Vesicle release rate	1 ms^−1^·M^−3^	[11]
$k_{3}$	Vesicle recovery rate	10 ms^−1^	[11]
nb	Ca^2+^ binding sites	4	[11]
$T_{\max}$	Glutamate concentration per vesicle	40 mM	[11]
$U_{0}$	Resting probability of glutamate release	0.3	[28]
$\alpha_{mGluR}$	mGluR-R activation rate	1500 × 10^3^ M^−1^ s^−1^	[28]
$\beta_{mGluR}$	mGluR-R inactivation rate	0.2 s^−1^	[28]
$\alpha_{GABAB}$	GABA_B_-R activation rate	1600 × 10^3^ M^−1^ s^−1^	Adapted from [28]
$\beta_{GABAB}$	GABA_B_-R inactivation rate	6 s^−1^	[28]
conv	Conversion factor from µA cm^−2^ to A m^−2^	0.01	–
$z_{Na}$	Na^+^ valency	1	–
$z_{K}$	K^+^ valency	1	–
$z_{Ca}$	Ca^2+^ valency	2	–

**Table A3:** Initial values of the presynaptic neuron compartment.

Variable	Description	Initial Value	Source
V	Pre membrane potential	−70 mV	[11]
Na	Na^+^ in Pre	15 mM	[11]
K	K^+^ in Pre	100 mM	[11]
Ca	Ca^2+^ in Pre	50 nM	[11]
$w\in \left\{m,h,n\right\}$	Gating variables for Na^+^ and K^+^ in a Hodgkin-Huxley formalism	${\left.\frac{\alpha_{w}}{\alpha_{w}+\beta_{w}}\right|}_{t=0}$	[11]
$m_{Ca}$	Gating variable for Ca^2+^	${\left.m_{Ca_{\inf}}\right|}_{t=0}$	[11]
Q	Concentration of activated synaptic vesicles	0.01 mM	[11]
RR	Rate of glutamate release	0	[11]
$r_{y}$, where $y\in \left\{GABAA,GABAB,mGluR\right\}$	Fractions of activated GABA_A_-Rs, GABA_B_-Rs and mGlu-Rs	0	[25,28]

**Table A4:** Variables from other modules connected to the presynaptic neuron compartment.

Variable	Description
$I_{app}$	External pulsed current applied to the Pre. Characteristics: amplitude 10 µM·cm^−2^, frequency 10 Hz, pulse width 3 ms. Necessary for Pre excitation.
$Na_{ecs}$	Na^+^ in ECS
$K_{ecs}$	K^+^ in ECS
$Ca_{ecs}$	Ca^2+^ in ECS
$Glu_{ecs}$	Glutamate in ECS
$GABA_{ecs}$	GABA in ECS

## Appendix B. Postsynaptic Neuron Compartment

**Table A5:** Equations of the postsynaptic neuron compartment.

Equation	Description	Source
$C\frac{dV}{dt}=I_{app_{delayed}}-\left(I_{Na}+I_{K}+I_{Ca}+I_{L}+I_{GABAA}\right)$	Change in membrane potential	Adapted from [11,25]
$I_{Na_{vg}}=g_{Na}m^{3}h\left(V-E_{Na}\right)$	Voltage-gated Na^+^ channels current	[11]
$I_{K_{vg}}=g_{K}n^{4}\left(V-E_{K}\right)$	Voltage-gated K^+^ channels current	[11]
$I_{Ca_{vg}}=g_{Ca}m_{Ca}^{3}\left(V-E_{Ca}\right)$	Voltage-gated Ca^2+^ channels current	[11]
$I_{L}=g_{L}\left(V-E_{L}\right)$	Background leak channels current	[11]
$\frac{dw}{dt}=\alpha_{w}\left(1-w\right)-\beta_{w}w$, where $w\in \left\{m,h,n\right\}$	Changes in gating variables for Na^+^ and K^+^ in a Hodgkin-Huxley formalism	[11]
$\frac{dm_{Ca}}{dt}=\frac{m_{Ca_{\inf}}-m_{Ca}}{\tau_{m_{Ca}}}$	Change in gating variable for Ca^2+^	[11]
$\alpha_{m}=\frac{2.5-0.1\left(V-V_{rest}\right)}{\exp\left(2.5-0.1\left(V-V_{rest}\right)\right)-1}$	Voltage-gated Na^+^ channel activation forward rate	[11]
$\beta_{m}=4\exp\left(\frac{V_{rest}-V}{18}\right)$	Voltage-gated Na^+^ channel activation backward rate	[11]
$\alpha_{h}=0.07\exp\left(\frac{V_{rest}-V}{20}\right)$	Voltage-gated Na^+^ channel inactivation forward rate	[11]
$\beta_{h}=\frac{1}{1+\exp\left(3-0.1\left(V-V_{rest}\right)\right)}$	Voltage-gated Na^+^ channel inactivation backward rate	[11]
$\alpha_{n}=\frac{0.1-0.01\left(V-V_{rest}\right)}{\exp\left(1-0.1\left(V-V_{rest}\right)\right)-1}$	Voltage-gated K^+^ channel activation forward rate	[11]
$\beta_{n}=0.125\exp\left(\frac{V_{rest}-V}{80}\right)$	Voltage-gated K^+^ channel activation backward rate	[11]
$m_{Ca_{\inf}}=\frac{1}{1+\exp\left(-\frac{V+24.758}{8.429}\right)}$	Voltage-gated Ca^2+^ channel equilibrium activation rate	[11]
$\tau_{m_{Ca}}=\left\{\begin{array}{l} 0.2702+1.1622\exp\left(\frac{-{\left(V+22.098\right)}^{2}}{164.19}\right),\mathrm{if}\;V\geq -40\;,\\ 0.6923\exp\left(\frac{V-4.7}{1089.372}\right),\;\;\mathrm{otherwise}. \end{array}\right.$	Voltage-gated Ca^2+^ channel activation time	[11]
$I_{Na}=I_{Na_{vg}}\cdot conv+3I_{NKA}+I_{AMPA}+I_{NMDA}+I_{NaL}$	Na^+^ membrane current density	Adapted from [11]
$I_{K}=I_{K_{vg}}\cdot conv-2I_{NKA}-I_{NMDA}+I_{KL}$	K^+^ membrane current density	Adapted from [11]
$I_{Ca}=I_{Ca_{vg}}\cdot conv+I_{PMCA}+I_{NMDA}+I_{CaL}$	Ca^2+^ membrane current density	Adapted from [11]
$I_{X_{post}}=I_{X}SA_{post}$, where $X\in \left\{Na,K,Ca\right\}$	Na^+^, K^+^ and Ca^2+^ membrane currents	[11]
$I_{AMPA}=g_{AMPA}r_{AMPA}\left(V-E_{AMPA}\right)$	AMPA-R current	[11]
$\frac{dr_{AMPA}}{dt}=\alpha_{AMPA}Glu_{ecs}\left(1-r_{AMPA}\right)-\beta_{AMPA}r_{AMPA}$	Change in fraction of activated AMPA-Rs	[11]
$I_{NMDA}=g_{NMDA}r_{NMDA}Mg_{V}\left(V-E_{NMDA}\right)$	NMDA-R current	[11]
$\frac{dr_{NMDA}}{dt}=\alpha_{NMDA}Glu_{ecs}\left(1-r_{NMDA}\right)-\beta_{NMDA}r_{NMDA}$	Change in fraction of activated NMDA-Rs	[11]
$Mg_{V}=\frac{1}{1+\frac{\exp\left(-0.062V\right)\cdot Mg_{ecs}}{3.57}}$	Magnesium block of NMDA-Rs	[11]
$I_{GABAA}=g_{GABAA}r_{GABAA}\left(V-E_{GABAA}\right)$	GABA_A_-R current	[25]
$\frac{dr_{GABAA}}{dt}=\alpha_{GABAA}GABA_{ecs}\left(1-r_{GABAA}\right)-\beta_{GABAA}r_{GABAA}$	Change in fraction of activated GABA_A_-Rs	[25]
$I_{NKA}=I_{NKA_{\max}}F\frac{Na^{1.5}}{Na^{1.5}+{K_{dNa}}^{1.5}}\frac{K_{ecs}}{K_{ecs}+K_{dK}}$	NKA current	[11]
$I_{PMCA}=I_{PMCA_{\max}}F\frac{Ca}{K_{PMCA}+Ca}$	PMCA current	[11]
$I_{XL}=g_{XL}\left(V-E_{X}\right)$, where $X\in \left\{Na,K,Ca\right\}$	Na^+^, K^+^ and Ca^2+^ leak currents	[11]
$E_{X}=\frac{RT}{z_{X}F}\ln\left(\frac{X_{ecs}}{X}\right)$, where $X\in \left\{Na,K,Ca\right\}$	Equilibrium potentials for Na^+^, K^+^ and Ca^2+^	[11]
$J_{X_{post}}=-\frac{I_{X_{post}}}{z_{X}F\cdot Vol_{post}}$, where $X\in \left\{Na,K,Ca\right\}$	Na^+^, K^+^ and Ca^2+^ membrane fluxes	[11]
$\frac{dX}{dt}=J_{X_{post}}$, where $X\in \left\{Na,K,Ca\right\}$	Changes in Na^+^, K^+^ and Ca^2+^ concentrations	[11]

**Table A6:** Parameter values of the postsynaptic neuron compartment.

Parameter	Description	Value	Source
F	Faraday’s constant	96,485.33 C·mol^−1^	–
R	Ideal gas constant	8.3145 J·mol^−1^·K^−1^	–
T	Absolute temperature	310 K	–
C	Membrane capacitance	1 µF·cm^−2^	[11]
$Vol_{post}$	Post volume	0.014314 fl	[11]
$SA_{post}$	Post surface area	0.21206 µm^2^	[11]
$V_{rest}$	Post resting potential	−70 mV	[11]
$g_{Na}$	Maximal voltage-gated Na^+^ channels conductance	120 mS·cm^−2^	[11]
$g_{K}$	Maximal voltage-gated K^+^ channels conductance	36 mS·cm^−2^	[11]
$g_{Ca}$	Maximal voltage-gated Ca^2+^ channels conductance	0.1 mS·cm^−2^	[11]
$g_{L}$	Maximal leak channels conductance	0.3 mS·cm^−2^	[11]
$g_{AMPA}$	Maximal AMPA-Rs conductance	0.26 S·m^−2^	[11]
$E_{AMPA}$	Equilibrium potential for AMPA-Rs	0 mV	[11]
$\alpha_{AMPA}$	AMPA-R activation rate	1100 × 10^3^ M^−1^ s^−1^	[11]
$\beta_{AMPA}$	AMPA-R inactivation rate	190 s^−1^	[11]
$g_{NMDA}$	Maximal NMDA-Rs conductance	0.18 S·m^−2^	[11]
$E_{NMDA}$	Equilibrium potential for NMDA-Rs	0 mV	[11]
$\alpha_{NMDA}$	NMDA-R activation rate	72 × 10^3^ M^−1^ s^−1^	[11]
$\beta_{NMDA}$	NMDA-R inactivation rate	6.6 s^−1^	[11]
$Mg_{ecs}$	Magnesium in ECS	1 mM	[11]
$g_{GABAA}$	Maximal GABA_A_-Rs conductance	0.5 S·m^−2^	[25]
$E_{GABAA}$	Equilibrium potential for GABA_A_-Rs	−85 mV	[25]
$\alpha_{GABAA}$	GABA_A_-R activation rate	500 × 10^3^ M^−1^ s^−1^	[25]
$\beta_{GABAA}$	GABA_A_-R inactivation rate	720 s^−1^	[25]
$I_{NKA_{\max}}$	Maximal NKA velocity	1.12 × 10^−6^ mol·m^−2^ s^−1^	[11]
$K_{dNa}$	NKA Na^+^ affinity	0.01 M	[11]
$K_{dK}$	NKA K^+^ affinity	0.6 × 10^−3^ M	[11]
$I_{PMCA_{\max}}$	Maximal PMCA velocity	0.2 × 10^−6^ mol·m^−2^ s^−1^	[11]
$K_{PMCA}$	PMCA Ca^2+^ affinity	0.2 × 10^−6^ M	[11]
$g_{NaL}$	Na^+^ leak conductance	${\left.-\frac{I_{Na_{vg}}\cdot conv+3I_{NKA}+I_{AMPA}+I_{NMDA}}{V-E_{Na}}\right|}_{t=0}$	Calculated for model stability
$g_{KL}$	K^+^ leak conductance	${\left.-\frac{I_{K_{vg}}\cdot conv-2I_{NKA}-I_{NMDA}}{V-E_{K}}\right|}_{t=0}$	Calculated for model stability
$g_{CaL}$	Ca^2+^ leak conductance	${\left.-\frac{I_{Ca_{vg}}\cdot conv+I_{PMCA}+I_{NMDA}}{V-E_{Ca}}\right|}_{t=0}$	Calculated for model stability
conv	Conversion factor from µA cm^−2^ to A m^−2^	0.01	–
$z_{Na}$	Na^+^ valency	1	–
$z_{K}$	K^+^ valency	1	–
$z_{Ca}$	Ca^2+^ valency	2	–

**Table A7:** Initial values of the postsynaptic neuron compartment.

Variable	Description	Initial Value	Source
V	Post membrane potential	−70 mV	[11]
Na	Na^+^ in Post	15 mM	[11]
K	K^+^ in Post	100 mM	[11]
Ca	Ca^2+^ in Post	50 nM	[11]
$w\in \left\{m,h,n\right\}$	Gating variables for Na^+^ and K^+^ in a Hodgkin-Huxley formalism	${\left.\frac{\alpha_{w}}{\alpha_{w}+\beta_{w}}\right|}_{t=0}$	[11]
$m_{Ca}$	Gating variable for Ca^2+^	$m_{Ca_{\inf}}$	[11]
$r_{y}$, where $y\in \left\{AMPA,NMDA,GABAA\right\}$	Fractions of activated AMPA-Rs, NMDA-Rs and GABA_A_-Rs	0	[11,25]

**Table A8:** Variables from other modules connected to the postsynaptic neuron compartment.

Variable	Description
$I_{app_{delayed}}$	External pulsed current applied to the Post. Characteristics: amplitude 0.067 µM·cm^−2^, frequency 10 Hz, pulse width 3 ms. Applied with a 2 ms delay relative to the Pre. Necessary for removing the magnesium block in the Post.
$Na_{ecs}$	Na^+^ in ECS
$K_{ecs}$	K^+^ in ECS
$Ca_{ecs}$	Ca^2+^ in ECS
$Glu_{ecs}$	Glutamate in ECS
$GABA_{ecs}$	GABA in ECS

## Appendix C. Astrocytic Compartment

**Table A9:** Equations of the astrocytic compartment.

Equation	Description	Source
$I_{Na}=-3I_{EAAT}+3I_{NCX}+3I_{NKA}+2I_{GAT}+I_{NaL}$	Na^+^ membrane current density	Adapted from [11,25]
$I_{K}=I_{EAAT}-2I_{NKA}+I_{Kir}+I_{KL}$	K^+^ membrane current density	[11]
$I_{Ca}=-I_{NCX}+I_{CaL}$	Ca^2+^ membrane current density	[11]
$I_{GABA}=I_{GAT}+I_{GABAL}$	GABA membrane current density	[25]
$I_{Glu}=I_{EAAT}+I_{GluL}$	Glutamate membrane current density	[25]
$I_{EAAT}=V_{EAAT}eff_{EAAT}F\frac{Gl{u_{ecs}}^{n}}{{K_{EAAT}}^{n}+Gl{u_{ecs}}^{n}}$	EAAT-2 current	[11]
$I_{NCX}=I_{NCX_{\max}}\left({\left(\frac{Na}{Na_{ecs}}\right)}^{3}\exp\left(\frac{\gamma FV}{RT}\right)-\frac{Ca}{Ca_{ecs}}\exp\left(\frac{\left(\gamma -1\right)FV}{RT}\right)\right)$	NCX current	[11]
$I_{NKA}=I_{NKA_{\max}}F\frac{Na^{1.5}}{Na^{1.5}+{K_{dNa}}^{1.5}}\frac{K_{ecs}}{K_{ecs}+K_{dK}}$	NKA current	[11]
$I_{Kir}=g_{Kir}\sqrt{K_{ecs}}\left(V-E_{Kir}\right)$	Kir4.1 current	[11]
$E_{Kir}=\frac{RT}{z_{K}F}\ln\left(\frac{K_{ecs}}{K}\right)$	Equilibrium potential for Kir4.1	[11]
$I_{GAT}=g_{GAT}\left(V-E_{GAT}\right)$	GAT-3 current	[25]
$E_{GAT}=\frac{RT}{F}\ln\left(\frac{GABA_{ecs}}{GABA}{\left(\frac{Na_{ecs}}{Na}\right)}^{2}\frac{Cl_{ecs}}{Cl}\right)$	Equilibrium potential for GAT-3	[25]
$I_{X_{psc}}=I_{X}SA_{psc}$, where $X\in \left\{Na,K,Ca,GABA,Glu\right\}$	Na^+^, K^+^, Ca^2+^ GABA and glutamate membrane currents	[11]
$J_{X_{psc}}=-\frac{I_{X_{psc}}+I_{pf_{X}}CSA_{lf}}{z_{X}F\cdot Vol_{psc}}$, where $X\in \left\{Na,K,Ca\right\}$	Na^+^, K^+^ and Ca^2+^ membrane fluxes	[11]
$J_{X_{psc}}=-\frac{I_{X_{psc}}}{z_{X}F\cdot Vol_{psc}}$, where $X\in \left\{GABA,Glu\right\}$	GABA and glutamate membrane fluxes	Adapted from [11,25]
$I_{pf_{X}}=K_{pf}\frac{-E_{X_{ast}}}{l_{lf}}\exp\left(-\varphi +\frac{Q}{k_{B}T}\sqrt{\frac{Q\left(-E_{X_{ast}}\right)}{l_{lf}\pi \varepsilon }}\right)$, where $X\in \left\{Na,K,Ca\right\}$	Leaflet diffusion of Na^+^, K^+^ and Ca^2+^	[11]
$E_{X}=\frac{RT}{z_{X}F}\ln\left(\frac{X_{ecs}}{X}\right)$, where $X\in \left\{Na,K,Ca,GABA,Glu\right\}$	Equilibrium potentials relative to the ECS	Adapted from [11,25]
$E_{X_{ast}}=\frac{RT}{z_{X}F}\ln\left(\frac{X_{ast}}{X}\right)$, where $X\in \left\{Na,K,Ca\right\}$	Equilibrium potentials for ions relative to the intracellular space of the astrocyte	[11]
$I_{XL}=g_{XL}\left(V-E_{X}\right)$, where $X\in \left\{Na,K,Ca,GABA,Glu\right\}$	Leak currents	Adapted from [11,25]
$\frac{dX}{dt}=J_{X_{post}}$, where $X\in \left\{Na,K,Ca\right\}$	Changes in ion concentrations	[11]
$\frac{dX}{dt}=J_{X_{post}}-g_{deg_{X}}X$, where $X\in \left\{GABA,Glu\right\}$	Changes in neurotransmitter concentrations accounting for decay	Adapted from [11,25]

**Table A10:** Parameter values of the astrocytic compartment.

Parameter	Description	Value	Source
F	Faraday’s constant	96,485.33 C·mol^−1^	–
R	Ideal gas constant	8.3145 J·mol^−1^·K^−1^	–
T	Absolute temperature	310 K	–
Q	Elementary charge	1.6002 × 10^−19^ C	–
$k_{B}$	Boltzmann’s constant	1.38 × 10^−23^ J·K^−1^	–
$Vol_{psc}$	PsC volume	0.031416 fl	[11]
$SA_{psc}$	PsC surface area	0.23562 µm^2^	[11]
$CSA_{lf}$	Leaflet cross-sectional area	0.007854 µm^2^	[11]
$l_{lf}$	Leaflet length	2 µm	[11]
V	Astrocyte resting potential	−80.7 mV	[11]
$V_{EAAT}$	Maximal EAAT-2 velocity	3 × 10^−6^ mol·m^−2^·s^−1^	[11]
$eff_{EAAT}$	Average EAAT-2 efficiency	0.5	[11]
$K_{EAAT}$	EAAT-2 glutamate affinity	20 µM	[11]
$I_{NCX_{\max}}$	Maximal NCX current	1 A·m^−2^	[11]
$I_{NKA_{\max}}$	Maximal NKA velocity	3.36 × 10^−6^ mol·m^−2^ s^−1^	[11]
$K_{dNa}$	NKA Na^+^ affinity	0.01 M	[11]
$K_{dK}$	NKA K^+^ affinity	3.6 × 10^−3^ M	[11]
$g_{Kir}$	Maximal Kir4.1 channels conductance	144 S·m^−2^	[11]
$g_{GAT}$	Maximal GAT-3 conductance	150 S·m^−2^	Adapted from [25]
Cl	Chloride in the PsC	40 mM	[43]
$Cl_{ecs}$	Chloride in the ECS	135 mM	[43]
$K_{pf}$	Poole—Frankel channel constant	0.018 S·m^−1^	[11]
φ	Well activation energy	10 J	[11]
ε	Dynamic permittivity	8.142 × 10^−12^	[11]
$g_{deg_{Glu}}$	Glutamate degradation rate	10 s^−1^	Adapted from [12]
$g_{deg_{GABA}}$	GABA degradation rate	0.1 s^−1^	Adapted from [12]
$Na_{ast}$	Na^+^ in the astrocyte intracellular space	15 mM	[11]
$K_{ast}$	K^+^ in the astrocyte intracellular space	100 mM	[11]
$Ca_{ast}$	Ca^2+^ in the astrocyte intracellular space	100 nM	[11]
$z_{Na}$	Na^+^ valency	1	–
$z_{K}$	K^+^ valency	1	–
$z_{Ca}$	Ca^2+^ valency	2	–
$z_{Glu}$	Glutamate valency	−1	–

**Table A11:** Initial values of the astrocytic compartment.

Variable	Description	Initial Value	Source
Na	Na^+^ in PsC	15 mM	[11]
K	K^+^ in PsC	100 mM	[11]
Ca	Ca^2+^ in PsC	100 nM	[11]
GABA	GABA in PsC	4.5 mM	[43]
Glu	Glutamate in PsC	1.5 mM	[25]

**Table A12:** Variables from other modules connected to the astrocytic compartment.

Variable	Description
$Na_{ecs}$	Na^+^ in ECS
$K_{ecs}$	K^+^ in ECS
$Ca_{ecs}$	Ca^2+^ in ECS
$Glu_{ecs}$	Glutamate in ECS
$GABA_{ecs}$	GABA in ECS

## Appendix D. Extracellular Space Compartment

**Table A13:** Equations of the extracellular space compartment.

Equation	Description	Source
$I_{X_{ecs}}=I_{X_{dif_{ecs}}}-I_{X_{psc}}-I_{X_{post}}-I_{X_{pre}}$, where $X\in \left\{Na,K,Ca\right\}$	Na^+^, K^+^ and Ca^2+^ currents in the ECS	[11]
$I_{X_{ecs}}=-I_{X_{psc}}+X_{rel}$, where $X\in \left\{GABA,Glu\right\}$	GABA and glutamate currents in the ECS	Adapted from [11,25]
$I_{X_{dif_{ecs}}}=g_{ecs}\lambda E_{X_{ecs}}SA_{ecs_{dif}}$, where $X\in \left\{Na,K,Ca\right\}$	Na^+^, K^+^ and Ca^2+^ ECS diffusion	[11]
$J_{X_{ecs}}=-\frac{I_{X_{ecs}}}{z_{X}F\cdot Vol_{ecs}}$, where $X\in \left\{Na,K,Ca,GABA,Glu\right\}$	Na^+^, K^+^, Ca^2+^, GABA and glutamate fluxes	[11]
$E_{X_{ecs}}=\frac{RT}{z_{X}F}\ln\left(\frac{X}{X_{gecs}}\right)$, where $X\in \left\{Na,K,Ca\right\}$	Equilibrium potentials for ions relative to the GECS	[11]
$\frac{dX}{dt}=J_{X_{ecs}}$, where $X\in \left\{Na,K,Ca,GABA\right\}$	Changes in Na^+^, K^+^, Ca^2+^ and GABA concentrations	Adapted from [11,25]
$\frac{dGlu}{dt}=J_{Glu_{ecs}}-\frac{Glu}{k_{T}}$	Change in glutamate concentration accounting for decay	Adapted from [11,25]

**Table A14:** Parameter values of the extracellular space compartment.

Parameter	Description	Value	Source
F	Faraday’s constant	96,485.33 C·mol^−1^	–
R	Ideal gas constant	8.3145 J·mol^−1^·K^−1^	–
T	Absolute temperature	310 K	–
$Vol_{ecs}$	ECS volume	0.00786 fl	[11]
$SA_{ecs_{dif}}$	ECS diffusion surface area	0.015 µm^2^	[11]
$g_{ecs}$	Maximal diffusion conductance	1 S·m^−2^	[11]
λ	Conductance scaling factor	10	[11]
$Na_{gecs}$	Na^+^ in the GECS	135 mM	[11]
$K_{gecs}$	K^+^ in the GECS	4 mM	[11]
$Ca_{gecs}$	Ca^2+^ in the GECS	1.5 mM	[11]
$k_{T}$	Glutamate decay rate	5 ms	[11]
$z_{Na}$	Na^+^ valency	1	–
$z_{K}$	K^+^ valency	1	–
$z_{Ca}$	Ca^2+^ valency	2	–
$z_{Glu}$	Glutamate valency	−1	–

**Table A15:** Initial values of the extracellular space compartment.

Variable	Description	Initial Value	Source
Na	Na^+^ in the ECS	135 mM	[11]
K	K^+^ in the ECS	4 mM	[11]
Ca	Ca^2+^ in the ECS	1.5 mM	[11]
GABA	GABA in the ECS	0.8 µM	[43]
Glu	Glutamate in the ECS	0.1 nM	[25]

**Table A16:** Variables from other modules connected to the extracellular space compartment.

Variable	Description
$I_{X_{pre}}$, where $X\in \left\{Na,K,Ca\right\}$	Na^+^, K^+^ and Ca^2+^ transmembrane Pre currents
${Glu}_{rel}$	Glutamate released by the Pre
$GABA_{rel}$	Glutamate released by the interneuron
$I_{X_{post}}$, where $X\in \left\{Na,K,Ca\right\}$	Na^+^, K^+^ and Ca^2+^ transmembrane Post currents
$I_{X_{psc}}$, where $X\in \left\{Na,K,Ca,GABA,Glu\right\}$	Na^+^, K^+^, Ca^2+^, GABA and glutamate transmembrane astrocytic currents

## Appendix E. Interneuron Compartment

**Table A17:** Equations of the interneuron compartment.

Equation	Description	Source
$C\frac{dV}{dt}=I_{app}-\left(I_{Na}+I_{K}+I_{L}\right)$	Change in membrane potential	[12]
$I_{Na_{vg}}=g_{Na}m^{3}h\left(V-E_{Na}\right)$	Voltage-gated Na^+^ channels current	[11]
$I_{K_{vg}}=g_{K}n^{4}\left(V-E_{K}\right)$	Voltage-gated K^+^ channels current	[11]
$I_{L}=g_{L}\left(V-E_{L}\right)$	Background leak channels current	[11]
$\frac{dw}{dt}=\alpha_{w}\left(1-w\right)-\beta_{w}w$, where $w\in \left\{m,h,n\right\}$	Changes in gating variables for Na^+^ and K^+^ in a Hodgkin-Huxley formalism	[11]
$\alpha_{m}=\frac{0.1\left(-V-45\right)}{\exp\left(\frac{-V-45}{10}\right)-1}$	Voltage-gated Na^+^ channel activation forward rate	[12]
$\beta_{m}=4\exp\left(\frac{-V-70}{18}\right)$	Voltage-gated Na^+^ channel activation backward rate	[12]
$\alpha_{h}=0.07\exp\left(\frac{-V-70}{20}\right)$	Voltage-gated Na^+^ channel inactivation forward rate	[12]
$\beta_{h}=\frac{1}{1+\exp\left(\frac{-V-40}{10}\right)}$	Voltage-gated Na^+^ channel inactivation backward rate	[12]
$\alpha_{n}=\frac{0.01\left(-V-60\right)}{\exp\left(\frac{-V-60}{10}\right)-1}$	Voltage-gated K^+^ channel activation forward rate	[12]
$\beta_{n}=0.125\exp\left(\frac{-V-70}{80}\right)$	Voltage-gated K^+^ channel activation backward rate	[12]
$\frac{dR}{dt}=\frac{I}{\tau_{rec}}-R\cdot \delta \left(t-t_{sp}\right)$	Change in fraction of synaptic vesicles in the recovered state	[12]
$\frac{dE}{dt}=-\frac{E}{\tau_{inact}}+R\cdot \delta \left(t-t_{sp}\right)$	Change in fraction of synaptic vesicles in the active state	[12]
I=1−R−E	Fraction of synaptic vesicles in the inactive state	[12]
$GABA_{rel}=n_{\nu }g_{\nu }E$	Concentration of GABA released per second	Adapted from [12]

**Table A18:** Parameter values of the interneuron compartment.

Parameter	Description	Value	Source
C	Membrane capacitance	1 µF·cm^−2^	[12]
$V_{rest}$	Interneuron resting potential	−70 mV	[12]
$g_{Na}$	Maximal voltage-gated Na^+^ channels conductance	120 mS·cm^−2^	[12]
$g_{K}$	Maximal voltage-gated K^+^ channels conductance	36 mS·cm^−2^	[12]
$g_{L}$	Maximal leak channels conductance	0.3 mS·cm^−2^	[12]
$E_{Na}$	Equilibrium potential for Na^+^	45 mV	[12]
$E_{K}$	Equilibrium potential for K^+^	−82 mV	[12]
$\tau_{rec}$	Vesicle recovery time constant	0.8 s	[12]
$\tau_{inac}$	Vesicle inactivation time constant	0.003 s	[12]
$n_{\nu }$	Number of docked vesicles	7	Adapted from [12]
$g_{\nu }$	GABA concentration in single vesicle	100 mM	Adapted from [12]

**Table A19:** Initial values of the interneuron compartment.

Variable	Description	Initial Value	Source
V	Interneuron membrane potential	−70 mV	[12]
m	Na^+^ gating variable in a Hodgkin-Huxley formalism (activation)	0.1	[12]
h	Na^+^ gating variable in a Hodgkin-Huxley formalism (inactivation)	0.6	[12]
n	K^+^ gating variable in a Hodgkin-Huxley formalism (activation)	0.3	[12]
R	Fraction of synaptic vesicles in the recovered state	1	[12]
E	Fraction of synaptic vesicles in the active state	0	[12]
